# Plasticity of circadian and circatidal rhythms in activity and transcriptomic dynamics in a freshwater snail

**DOI:** 10.1038/s41437-024-00680-7

**Published:** 2024-03-27

**Authors:** Takumi Yokomizo, Yuma Takahashi

**Affiliations:** Graduate School of Science, Chiba University, Chiba, 263-8522 Japan

**Keywords:** Molecular ecology, Evolutionary ecology

## Abstract

Organisms have diverse biological clocks synchronised with environmental cycles depending on their habitats. Anticipation of tidal changes has driven the evolution of circatidal rhythms in some marine species. In the freshwater snail, *Semisulcospira reiniana*, individuals in nontidal areas exhibit circadian rhythms, whereas those in tidal areas exhibit both circadian and circatidal rhythms. We investigated whether the circatidal rhythms are genetically determined or induced by environmental cycles. The exposure to a simulated tidal cycle did not change the intensity of circatidal rhythm in individuals in the nontidal population. However, snails in the tidal population showed different activity rhythms depending on the presence or absence of the exposure. Transcriptome analysis revealed that genes with circatidal oscillation increased due to entrainment to the tidal cycle in both populations and dominant rhythmicity was consistent with the environmental cycle. These results suggest plasticity in the endogenous rhythm in the gene expression in both populations. Note that circatidal oscillating genes were more abundant in the tidal population than in the nontidal population, suggesting that a greater number of genes are associated with circatidal clocks in the tidal population compared to the nontidal population. This increase of circatidal clock–controlled genes in the tidal population could be caused by genetic changes in the biological clock or the experience of tidal cycle in the early life stage. Our findings suggest that the plasticity of biological rhythms may have contributed to the adaptation to the tidal environment in *S. reiniana*.

## Introduction

Organisms have faced the challenge of adapting to periodic variations in environments since ancient times. The rotation and inertial force of Earth, along with the gravitational forces of the sun and moon, generate environmental variations across multiple timescales, including day–night, tidal, lunar, and seasonal cycles. To cope with these fluctuations, organisms possess self-sustained, temperature-compensated timekeeping systems capable of entraining to environmental cues (Dunlap 1999; Bell-Pedersen et al. 2005; Yerushalmi and Green 2009). The circadian rhythm, observed in a wide range of organisms, ranging from bacteria (Kondo et al. 1993) to mammals (Takahashi, 1995; King and Takahashi 2000; Reppert and Weaver 2001), allows for adaptation to day–night cycles. Biological clocks enable organisms to anticipate environmental changes and regulate physiological processes with appropriate timing. The coordination between environmental cycles and biological clock parameters, such as phase and period, plays a vital role in enhancing fitness and facilitating adaptation to rhythmic environments (Yerushalmi et al. 2011; Rubin et al. 2017, 2018).

Synchronising biological rhythms with environmental cycles has adaptive significance and can result in variations in endogenous rhythms depending on the circumstances. For instance, studies on the model fungus *Neurospora discreta* have shown that local adaptation to different rhythmic environments, regulated by the circadian clock, leads to habitat-specific endogenous rhythms and influences reproductive success (Koritala et al. 2020). Variations in endogenous rhythms can arise not only from local adaptation but also from plasticity driven by social roles. Honeybees, for example, exhibit social environment-dependent plasticity in their circadian rhythm within a single population (Bloch and Robinson 2001; Shemesh et al. 2007). These findings suggest that genetic and/or nongenetic changes in biological rhythms contribute to enhancing individual performance.

Organisms residing in marine or intertidal habitats encounter substantial and complex environmental changes, including fluctuations in water level, salinity, temperature, light, and food availability, driven by daily and tidal cycles. The circatidal rhythm, an approximately 12-h biological rhythm synchronised with tides, is observed in diverse taxa (Barnwell 1966; Satoh et al. 2008; Tessmar-Raible et al. 2011; Chabot et al. 2016). Owing to the critical importance of anticipating tidal changes for marine species, the selective pressure exerted by the tidal cycle can drive the adaptive evolution of circatidal clocks. The relationship between circadian and circatidal rhythms, characterised by different periodicities and zeitgebers, remains controversial. For example, studies on the mangrove cricket suggest that the circatidal timekeeping system operates independently of circadian clocks (Takekata et al. 2012, 2014). This dissociation in the circadian and circatidal clocks of mangrove crickets may be attributed to the adaptive evolution of a novel timekeeping system that emerged in their mangrove habitat. Conversely, investigations of various marine species have demonstrated plasticity in rhythm expression within individuals or populations under different environmental conditions (Mat et al. 2014; O’Neill et al. 2015; Schnytzer et al. 2018). Notably, certain circadian clock genes in the Pacific oyster *Crassostrea gigas* exhibit oscillation at tidal frequencies under tidal conditions (Mat et al. 2016; Tran et al. 2020). In the amphipod crustacean *Parhyale hawaiensis*, the core circadian clock gene *Bmal1* is suggested to be essential for circatidal rhythms (Kwiatkowski et al. 2023). These studies give rise to the hypothesis that the circadian and circatidal rhythms may be generated by a single biological timekeeping system, with circatidal clocks sharing some molecular components with circadian clocks. The expression of circatidal rhythms and successful adaptation to tidal environments likely involve a combination of plasticity and genetic changes.

Rivers present complex rhythmic environments due to diurnal and tidal cycles. Although a river follows a 24-h light–dark (LD) cycle throughout its course, downstream areas experience an environmental cycle with a period of 12.4 h due to the tidal cycle. To investigate variations in endogenous rhythm across habitats with distinct environmental cycles, we focused on the freshwater snail *Semisulcospira reiniana*, which inhabits both nontidal and tidal areas of a river. Our previous study revealed that snails in nontidal areas exhibit circadian rhythms, whereas those in tidal areas exhibit both circadian and circatidal rhythms (Yokomizo and Takahashi 2022). However, it remains unclear whether the differential rhythmicity arises from evolutionary differentiation or plasticity in response to environmental cycles. In the present study, we evaluated genetic and nongenetic changes in the endogenous rhythm of snails living in habitats characterised by heterogeneous environmental cycles. We exposed snails to a simulated tidal environment in the laboratory and examined the activity and transcriptome rhythms of individuals from both tidal and nontidal populations.

## Materials and methods

### Species and sampling sites

*Semisulcospira reiniana*, a common freshwater snail species found in Japan, was collected from nontidal (3–5 m above sea level; 35° 15′ 15″ N, 136° 41′ 09″ E) and tidal areas (<1 m above sea level; 35° 08′ 49″ N, 136° 40′ 37″ E) of the Kiso River in June and November 2021. The two populations were approximately 20 km apart along the river, and thus, snails of these populations were unlikely to migrate to each other. The collected snails were acclimated in the laboratory under 23 °C and 12–12-h LD (12L12D) conditions in freshwater for at least one month. The animal study was reviewed and approved by the ethics committee of Chiba University (No. DS1-3).

### Entrainment to the simulated tidal cycle

After acclimation, snails from the tidal and nontidal populations were divided into control and treatment groups. Individuals in the control group were kept under 23 °C and 12L12D conditions (light was on at 6:00 and off at 18:00) in freshwater without water level oscillation. The treatment group individuals were exposed to a simulated tidal cycle by raising and lowering the water level in a water tank (60 × 30 × 45 cm; Fig. S1). Due to the limitation of the capacity of the tank, the tidal simulations for behavioural observation and transcriptome analysis were conducted on separate dates. Containers (φ7 × 9 cm) with water vents were placed 32 cm from the bottom of the tank. Four snails were placed in each container. For the high tide condition, the water level was filled to a depth of 39 cm, submerging the snails completely. To simulate the low tide condition, the water level was dropped to a depth of 24 cm, exposing the snails completely to air. To emphasise the entrainment to tidal conditions independent of the diurnal cycle, water supply and draining were controlled by timers so that the time of low and high tides differed between the two simulations. In the simulation for the behavioural observation, water supply switched to drainage at 03:00 and 15:00, and drainage switched to supply at 09:00 and 21:00. In the simulation for transcriptome analysis, water supply switched to drainage at 05:00 and 17:00, and drainage switched to supply at 11:00 and 23:00. The entrainment treatment lasted for four weeks. The diurnal and temperature conditions were the same for the control group.

### Behavioural observation

To examine the effect of 12-h water level oscillation on the endogenous activity rhythm of *S. reiniana*, individuals from the control and treatment groups in both nontidal and tidal populations were observed under constant laboratory conditions. Eight snails per population per condition (32 individuals) were individually placed in containers (13 × 11.7 cm, 4.7 cm in height) filled with freshwater. The snails were observed under constant darkness (DD) at a temperature of 23 °C in the laboratory for 96 and 84 h in the control and treatment groups, respectively. A camera malfunction resulted in a shorter observation period for the treatment group compared with the control group. Water circulation was maintained using a pump to keep the water clean. Images were captured every 30 s using a model 400-CAM061 camera (Sanwa Direct, Japan) and were used to create time-lapse movies. UMATracker tracking software (Yamanaka and Takeuchi 2018) was used to track the positions of snails. After correcting major tracking errors, the coordinates of the snails that survived until the end of the observation were averaged every three frames to minimise the influence of small deviations. The total locomotion distance travelled in 1 h was calculated using the snail trajectories.

### RNA sampling

To investigate the effect of a 12-h water level oscillation on the endogenous gene expression rhythm of *S. reiniana*, time-course RNA sequencing (RNA-seq) was conducted using individuals from the control and treatment groups in both nontidal and tidal populations. Snails were preserved in containers without water under the DD condition at a constant temperature (23 °C) for tissue collection. Starting at subjective high tide, three individuals per population were dissected every 3.1 h for a total of 49.6 h (17 sampling time points) in both control and treatment groups and the foot and inner tissues were preserved in 750 μL of RNA*later* Stabilisation Solution (Invitrogen). The samples were stored at 4 °C for approximately 3 h and then stored at −80 °C until total RNA extraction.

### RNA extraction and sequencing

Total RNA was extracted using the Maxwell 16 LEV Plant RNA Kit, suitable for organisms containing polysaccharides such as molluscs, with the Maxwell 16 Research Instrument (Promega, USA) following the manufacturer’s instructions. Because a sufficient amount and quality of RNA was not extracted from the inner tissue, the foot tissue was used for RNA extraction and the rhythms of its peripheral clocks were analysed. Electrophoresis was performed on a 1% agarose gel to assess RNA degradation. RNA concentrations were estimated using the Qubit 2.0 fluorometer (Invitrogen), and RNA purity was assessed using the NanoDrop Lite Spectrophotometer (Thermo Scientific). Total RNA from three individuals in the same population at each sampling time point was pooled in equal amounts for pooled RNA-seq. Although we had no biological replicates per sampling time point, variations among individuals could be homogenised by pooled sequencing. A cDNA library was constructed using the TruSeq RNA Sample Prep Kit, and paired-end (150 bp) pooled RNA-seq was performed on the Illumina NovaSeq6000 platform. Adaptor sequences and low-quality reads were removed using Trimmomatic (version 0.38) (Bolger et al. 2014), and FastQC (version 0.11.8; http://www.bioinformatics.babraham.ac.uk/projects/fastqc/) was used for quality control. The remaining high-quality reads from all samples were used for de novo assembly via Trinity software (version 2.9.1) (Grabherr et al. 2011) with default parameters. To estimate gene expression levels, reads from each sample were mapped to the reference transcripts using RSEM software (version 1.3.0) (Li and Dewey 2014) with parameters –*est_method* RSEM, –*aln_method* bowtie2, –*trinity_mode*, and –*prep_reference* to obtain the transcripts per million (TPM) for each gene. The reference transcripts were used to create supertranscripts, and a BLAST search was performed against all protein sequences of *C. gigas* (Peñaloza et al. 2021) to identify homologues for each gene with the best hit and an e-value < 0.0001. Genes showing circatidal oscillation were analysed based on the identified homologues.

### Statistical analyses

The activity rhythm of individuals in the control and treatment groups was examined using Lomb–Scargle periodogram analysis via ActogramJ, a software package based on ImageJ for chronobiological data analysis and visualisation (Schmid et al. 2011). The locomotor distance of each survived individual was used for rhythm detection. Owing to the limited movement of individuals in the treatment group at the end of the observation, activity data from 72 h after the onset were used for the periodogram analysis. The max power (the intensity of period) in the circadian (20–28 h) and circatidal (10.4–14.4 h) range was calculated in Lomb–Scargle periodogram and analysed by the generalised linear model (GLM) with a Gamma distribution. For transcriptome analysis, genes with low expression (average TPM ≤ 1) or small oscillation amplitude (peak/trough ≤1.3) were excluded. Probabilistic principal component analysis (PCA) was performed to summarise the gene expression pattern of each sample. After removing an outlier sample, Tukey’s honestly significant difference (HSD) test was conducted to identify differences in expression patterns between populations or entrainment treatments. Gene expression rhythmicity was analysed using RAIN (Thaben and Westermark 2014), a nonparametric algorithm for identifying rhythmic components in large biological datasets. Circatidal (12.4 ± 3.1 h) and circadian (24.8 ± 3.1 h) oscillating genes (*p* < 0.01) were identified. A differential rhythmicity score (*S*_DR_) was calculated for each gene to assess the differential rhythmicity of gene expression between the control and treatment groups. Based on periodicity analysis performed via RAIN, genes with a *P*-value of circatidal periodicity <1 were used for the calculation of *S*_DR_, which was defined as follows:

$${S}_{{DR}}=\frac{{Z}_{P}+{Z}_{R}}{\sqrt{2}},$$

where *Z*_*P*_ and *Z*_*R*_ represent the *Z*-scores for changes in periodicity and amplitude between groups, respectively (Kuintzle et al. 2017). Changes in periodicity were calculated as log (*P* value of the control group)−log (*P* value of the treatment group), whereas changes in amplitude were calculated as log_2_ (amplitude of the treatment group / amplitude of the control group). The amplitude of each gene expression was defined as the difference between the maximum and minimum TPM values. The *P*-value for *S*_DR_ was computed using a Gaussian distribution fitted to the empirical distribution, and it was adjusted using the Benjamini & Hochberg procedure for multiple testing. To assess the relative significance of circadian and circatidal rhythms in the transcriptome, the log-scaled ratio of the *P* value for circadian periodicity to that for circatidal periodicity was calculated for each gene. Functional enrichment analysis of biological processes in circatidal oscillating genes or differential rhythmic genes based on *S*_DR_ was performed using Kyoto Encyclopedia of Genes and Genomes (KEGG) pathway over-representation analysis and KEGG gene set enrichment analysis (KEGG-GSEA) conducted through clusterProfiler (Yu et al. 2012). Circatidal oscillating genes identified via RAIN in the treatment group were used as the test set for KEGG pathway over-representation analysis. The gene list for GSEA was generated based on *S*_DR_. All statistical analyses were conducted using R version 4.2.2.

## Results

### Activity rhythm

**Fig. 1:**
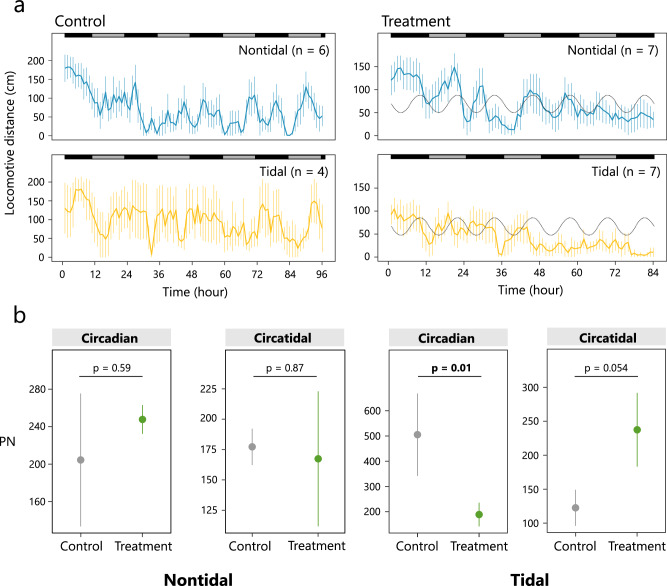
Activity patterns of individuals in the nontidal and tidal populations of the control group under laboratory DD conditions. **a** Mean locomotor distance of individuals in the control (left panels) and the treatment (right panels) of the nontidal (upper panels) and tidal (lower panels) populations. Error bars represent the standard error of the mean (SEM). Subjective day and night are indicated by grey and black bars above the patterns, respectively. The expected tide level in the laboratory is shown as a grey dotted line. **b** Mean power of Lomb–Scargle periodogram analysis for each individual. Error bars represent the SEM.

Snails exhibited rhythmic activity patterns under DD conditions in the laboratory, with a gradual decrease in activity observed in the treatment group (Fig. 1a). In individuals from the nontidal population, neither the power of the circadian period nor that of the circatidal period was significantly different between the control and treatment groups (circadian: χ^2^ = 0.30, *P* = 0.59; circatidal: χ^2^ = 0.03, *P* = 0.87, Fig. 1b). In contrast, individuals from the tidal population showed the power of periodicity corresponding to environmental cycles. The power of the circadian period in the control group was significantly higher than that of the treatment group (χ^2^ = 6.17, *P* = 0.01). The power of the circatidal period in the treatment group was higher compared to that of the control group, although the difference between them was marginally significant (χ^2^ = 3.70, *P* = 0.054).

### Transcriptome rhythm

**Fig. 2:**
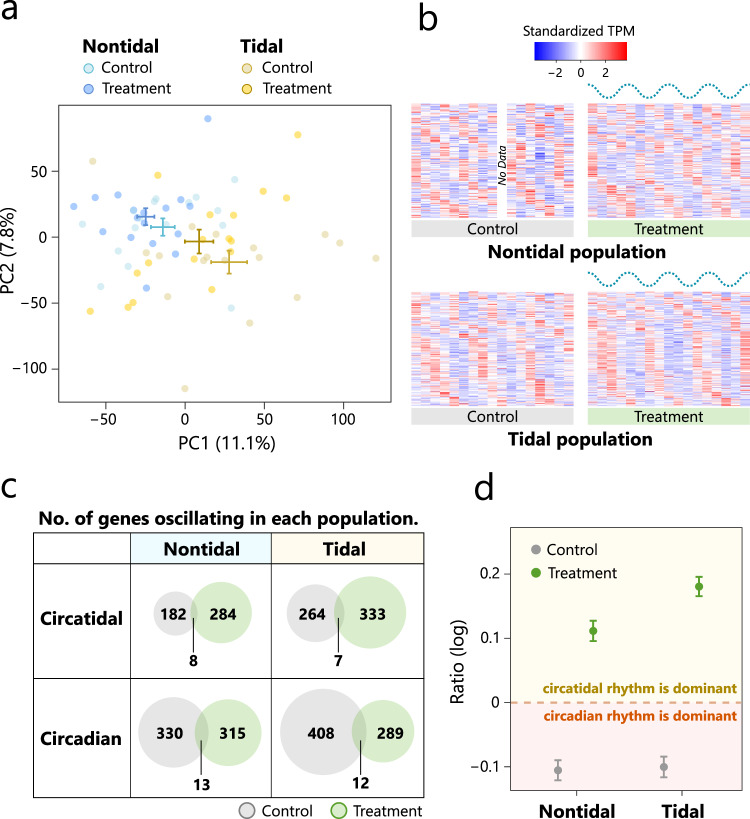
Gene expression patterns and rhythms of individuals in the nontidal and tidal populations under laboratory DD conditions. Control samples were kept under LD conditions without the tidal cycle. Treatment samples were exposed to the simulated tidal cycle for four weeks. **a** Probabilistic PCA of the expression of all genes after filtering in the nontidal and tidal populations. **b** Heatmaps of standardised expression patterns of rhythmic transcripts with circatidal periods in the nontidal and tidal individuals under DD conditions detected via RAIN. The dotted wavy lines above the heatmaps of treatment samples represent the simulated tide level. **c** Venn diagrams detailing the number of circatidal and circadian oscillating genes detected in the nontidal and tidal populations. **d** The dominant rhythmicity of the transcriptome in the control and treatment groups of the nontidal and tidal populations evaluated by log-scaled ratio of the *P* value of circadian periodicity to that of circatidal periodicity in the transcriptome. The positive value of log-scaled ratio represents that the circatidal rhythm is dominant in the transcriptome and the negative value represents that the circadian rhythm is dominant. The dashed line represents the same *P* values for circadian and circatidal periodicity. Error bars represent the SEM.

Information on the number of reads in each sample was summarised in Table S1. In total, we obtained 1,578,392 contigs with a mean length of 513 bp. Among the 954 metazoan core gene orthologs, we identified 948 (99.3%). We obtained 908,817 supertranscripts containing 58,952 contigs annotated against all protein sequences of *C. gigas*. In the tidal population, 13,929 and 14,508 genes in the control group and treatment group, respectively, met the criteria of average TPM value > 0 and oscillation amplitude (peak/trough) ≥1.3. In the nontidal population, the corresponding numbers were 13,448 and 13,997 genes in the control group and treatment group, respectively. Based on probabilistic PCA, one sample in the control group of the nontidal population showed a greatly different expression pattern compared with the other samples (Fig. S2) and was excluded from subsequent analysis. Probabilistic PCA of the remaining samples indicated significant differences in expression patterns between populations but not between the control and treatment groups (Fig. 2a; Table 1). In the nontidal population, we identified 190 (1.4%) and 292 (2.1%) circatidal (12.4 ± 3.1 h) oscillating genes in the control group and treatment group, respectively (Fig. 2b, c; Dataset S1). In the tidal population, the corresponding numbers were 271 (1.9%) and 340 (2.3%) circatidal oscillating genes in the control group and treatment group, respectively (Fig. 2b, c; Dataset S1). The proportion of genes oscillating in the circatidal period increased in both populations with entrainment to the simulated tidal cycle. However, the abundance of oscillating genes was higher in the tidal population compared with the nontidal population, regardless of the control or treatment group. We also detected several oscillating genes with a circadian period. In the nontidal population, 343 (2.6%) and 328 (2.3%) oscillating genes were found in the control and treatment groups, respectively (Fig. 2b, c; Dataset S1). In the tidal population, the corresponding numbers were 420 (3.0%) and 301 (2.1%) in the control and treatment groups, respectively (Fig. 2b, c; Dataset S1). Unlike circatidal oscillating genes, the proportion of genes oscillating in the circadian period decreased in both populations with entrainment to the simulated tidal cycle. We found a few oscillating genes shared between populations or environmental conditions (Fig. S3). We also detected genes which changed their expression rhythms from circadian to circatidal period in each population in response to the dominant environmental cycle (Table S2). There was no overlap between two populations. Among genes which passed the filtering, no circadian clock genes showed circadian or circatidal rhythmicity in the control and treatment groups in both populations (Figs. S4 and S5).

**Table 1:** Tukey’s HSD test of differences in gene expression patterns based on PC1.

	Mean difference	*P* value
Nontidal vs. Tidal	37.67	4.7 × 10^−5^
Control vs. Treatment	−14.80	0.09
Nontidal Control vs. Nontidal Treatment	−10.74	0.82
Nontidal Control vs. Tidal Control	41.51	0.0067
Nontidal Control vs. Tidal Treatment	22.77	0.26
Nontidal Treatment vs. Tidal Control	−52.25	3.2 × 10^−4^
Nontidal Treatment vs. Tidal Treatment	33.51	0.036
Tidal Control vs. Tidal Treatment	−18.74	0.41

**Fig. 3:**
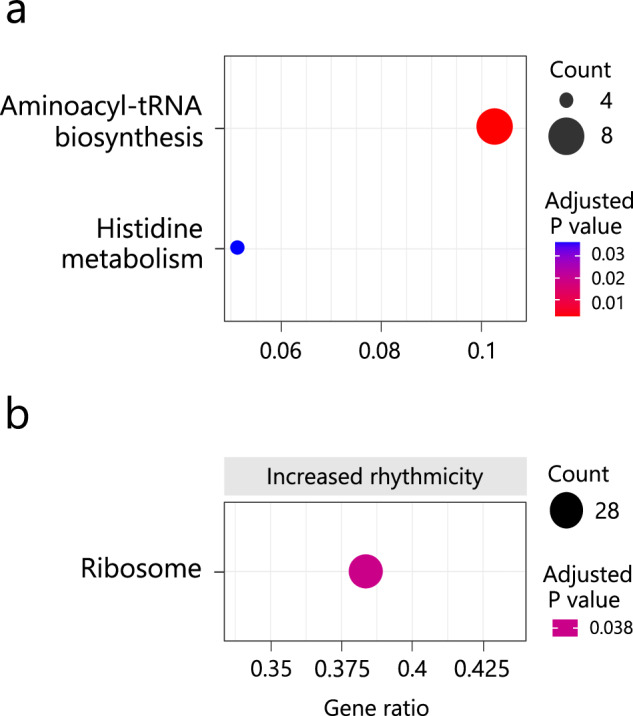
KEGG pathway analysis of the tidal population. **a** KEGG pathway over-representation analysis of the tidal population. Gene ratio refers to the ratio of the number of differentially expressed genes (DEGs) to the total number of genes in a specific pathway. The size of the circles represents the count of DEGs. Circles are coloured based on the adjusted *P* value. **b** KEGG-GSEA of the tidal population. Gene ratio refers to the ratio of the number of core enriched genes to the total number of genes in a specific pathway. The size of the circles represents the count of the core enriched genes. No pathways involved in decreased circatidal rhythmicity were detected.

Differential rhythmicity analysis revealed changes in transcriptome rhythmicity between the control and treatment groups (Fig. S6). Genes in the first quadrant exhibited clearer periodicity and larger amplitude in the treatment group compared with the control group, indicating increased circatidal rhythmicity. In the nontidal population, 13 genes showed significantly increased circatidal rhythmicity in the treatment group (FDR < 0.05; Table S3). In the tidal population, 16 genes showed significantly increased circatidal rhythmicity in the treatment group (FDR < 0.05; Table S4). Although most transcripts did not show significant differential rhythmicity in expression patterns in the tidal and nontidal populations, the log-scaled ratio of the *P* value for circadian periodicity to that for circatidal periodicity changed with entrainment to the tidal cycle (Fig. 2d). In both populations, the log-scaled *P* value ratio was negative in the control groups, indicating dominance of the circadian rhythm over the circatidal rhythm under LD conditions without a tidal cycle. However, the ratio was positive in the treatment groups, indicating dominance of the circatidal rhythm over the circadian rhythm after entrainment to the tidal cycle. KEGG pathway over-representation analysis revealed that circatidal oscillating genes in the treatment group of the tidal population were enriched for pathways of “Aminoacyl-tRNA biosynthesis” (KO: crg00970) and “Histidine metabolism” (KO: crg00340) (Fig. 3a). KEGG-GSEA based on *S*_DR_ revealed enrichment of the biological process “Ribosome” (KO: crg03010) for genes with increased circatidal rhythmicity in the tidal population (Fig. 3b). Based on both KEGG pathway over-representation analysis and KEGG-GSEA, no enriched pathways were detected in the nontidal population.

## Discussion

The coordination of biological processes with environmental cycles is regulated by biological clocks, such as the circadian clock, which enhances fitness in organisms in rhythmic environments (Sharma 2003). Marine and intertidal habitats, characterised by the tidal cycle, experience complex and drastic environmental variations, unlike terrestrial and inland water habitats. The freshwater snail *S*. *reiniana*, found in tidal areas, exhibits the circatidal rhythm, which is believed to be an adaptation to the tidal environment (Yokomizo and Takahashi 2022). In the present study, we investigated whether the differential rhythmicity in *S. reiniana* between tidal and nontidal populations is a result of evolutionary differentiation or plastic expression of rhythms depending on dominant environmental cycles. We demonstrated that only individuals in the tidal population exhibited a clear entrainment of the activity rhythm to the tidal cycle. Our results regarding the transcriptome rhythm suggest that more genes or pathways are influenced by the tidal cycle in the tidal population compared with the nontidal population, although circatidal oscillating genes increased in the treatment group, irrespective of population.

We first examined the influence of the simulated tidal cycle on the activity rhythm under constant conditions in each population. In the nontidal population, neither the intensity of circadian nor circatidal rhythms changed between the control and treatment groups, suggesting that snails in the nontidal population do not entrain their endogenous clocks to the tidal cycle. However, we found a large variation in the power of circatidal rhythm within the treatment group, indicating that a few individuals could show a moderate circatidal rhythm. Individuals in the tidal population changed the intensity of activity rhythm, although the difference was marginally significant for the circatidal rhythm, probably due to the small sample size. Our results suggest that water level fluctuations could serve as an environmental cue for modifying the endogenous rhythm and enhancing the circatidal rhythmicity in the tidal population.

Based on probabilistic PCA, we observed distinguishable gene expression patterns between populations rather than between the control and treatment groups, indicating differentiation in transcriptome expression between populations. Focusing on rhythmic transcripts, we found an increase in the number of circatidal oscillating genes in both populations following entrainment to the tidal cycle, accompanied by a decrease in the number of circadian oscillating genes. These results suggest that the circatidal rhythm in the expression level is plastically expressed in both populations, and the reduction in circadian oscillating genes supports the transition of expression of dominant endogenous rhythm depending on environmental cycles. Additionally, we identified a greater number of circatidal oscillating genes in the tidal population compared with the nontidal population. This result implies that the experience of tidal cycles in the early life stage or genetic changes in the biological clocks via adaptation to the tidal environment contribute to the increase in circatidal clock–controlled genes in individuals from the tidal population. We also detected hundreds of circatidal oscillating genes in the control group of nontidal populations. The expression rhythm with a period of 12.4 h in some of these genes might arise from the harmonics of circadian clocks, as observed in mammals (Hughes et al. 2009; Zhu et al. 2017; Zhu 2020). The small number of shared oscillating genes between populations or experimental conditions could suggest that the expression rhythms of genes controlled by clocks could easily be changed, and the response process to the tidal cycle could differ between populations. Although we identified several circadian clock genes, none of these genes exhibited circadian rhythmicity in their expression. This finding is consistent with previous studies that reported the absence of circadian rhythmic expression in clock genes in several intertidal organisms (Zhang et al. 2013; Schnytzer et al. 2018; Satoh and Terai 2019). The interference of circatidal rhythms with the rhythmic expression of circadian clock genes and weak peripheral clocks in the foot tissue or asynchronization between tissues in the samples could potentially explain the lack of rhythmic expression in these genes.

We found that a few genes changed the expression rhythms from the circadian to the circatidal period. This result suggests that a small number of genes respond to both light-dark and tidal cycles. To assess the influence of the tidal cycle on genes with weak oscillation in the transcriptome, we calculated the *S*_DR_ for each gene in both populations. The *S*_DR_ distribution in the tidal population was similar to that in the nontidal population, indicating comparable changes in circatidal rhythmicity. The number of genes showing a significant increase in circatidal rhythmicity did not differ substantially between populations. These results suggest that the degree of changes in circatidal rhythmicity in the transcriptome is similar in individuals exposed to the tidal cycle, regardless of population. Although the majority of genes did not exhibit significant changes in the strength of circatidal rhythmicity between the control and treatment groups, we identified 13 and 16 genes with significantly increased rhythmicity in the treatment group of the nontidal and tidal populations, respectively. These genes are likely entrained by the simulated tidal cycle, leading to enhanced circatidal rhythmicity. Additionally, we calculated the ratio of *P*-values for circadian periodicity to circatidal periodicity for each gene. Comparing the ratios between the control and treatment groups revealed that the dominant rhythmicity of the transcriptome aligns with the environmental cycle.

We conducted KEGG over-representation analysis and KEGG-GSEA to identify enriched biological processes among the circatidal oscillating genes and differential rhythmic genes. In the tidal population, we detected three pathways, whereas no pathways were identified in the nontidal population. Considering the larger number of circatidal oscillating genes in the tidal population, it is likely that individuals in the tidal population possess a higher abundance of circatidal clock–controlled genes and biological processes regulated by circatidal clocks compared with individuals in the nontidal population. The enrichment of the “Aminoacyl-tRNA biosynthesis” (KO: crg00970) and “Ribosome” (KO: crg03010) pathways in the circatidal oscillating genes of the treatment group in the tidal population suggests the involvement of circatidal rhythms in biological processes related to translation, as aminoacyl-tRNA is responsible for delivering amino acids for mRNA-guided protein synthesis at the ribosome. Furthermore, the detection of the “Histidine metabolism” (KO: crg00340) pathway suggests a potential association between proteins containing histidine and the response to the tidal cycle.

Our findings demonstrate the plasticity of gene expression rhythms in both populations, suggesting that the genetic basis of the circatidal rhythm may be conserved in the genome, with the tidal zeitgeber triggering the expression of the circatidal rhythm. This plasticity may be advantageous for individuals in nontidal areas to perceive slight environmental changes by the tidal force. However, the changes in circadian and circatidal activity rhythms were observed only in the tidal population. The plasticity of the endogenous rhythm and subsequent phenotypic differentiation by genetic changes likely played a role in the adaptation of *S. reiniana* to tidal areas in rivers. We have previously revealed minimal genetic differentiation between the two populations (Yokomizo and Takahashi 2022), supporting the contribution of plasticity and the evolution of a small number of genes to range expansion. By investigating other rivers where *S. reiniana* is limited to nontidal areas, we can further explore the relationship between the plasticity of the endogenous rhythm and the establishment in tidal areas. Although we found hundreds of genes oscillating in a circadian and circatidal period, it remains unclear whether snails possess independent circatidal clocks alongside circadian clocks or a shared timekeeping system between circadian and circatidal rhythms. The latter hypothesis is gaining support, particularly in studies on biological clocks in marine species (Schnytzer et al. 2018; Tran et al. 2020; Mat et al. 2020; Kwiatkowski et al. 2023). Further investigations are needed to elucidate the genetic mechanisms underlying circatidal clocks in freshwater snails. However, the absence or decrease of circadian rhythm in activity and gene expression after entrainment to the tidal cycle supports the notion that circadian and circatidal rhythms are not completely independent and may interact. Unravelling the molecular relationship between circadian and circatidal clocks would contribute to testing the debated hypotheses on the mechanism of circatidal oscillation generation (Enright 1976; Hastings and Naylor 1980; Palmer 1995).

Our study indicates that individuals in the nontidal population showed the circatidal rhythm in gene expression but not in activity when exposed to the tidal cycle. In contrast, transcriptome analysis suggests that individuals in the tidal population exhibit increased regulation through the circatidal clock. Overall, snails in both tidal and nontidal populations have the plasticity of the endogenous rhythm, but there is also differentiation in rhythmicity between populations. Our findings suggest that the plasticity and subsequent genetic assimilation may contribute to adaptation to the tidal environment. Investigating the parallel evolution of biological clocks using individuals from independent rivers would provide a comprehensive understanding of the mechanisms underlying adaptation to tidal environments, including genetic and nongenetic changes in biological clocks.

### Data archiving

All raw transcriptome read data were deposited in the DDBJ Sequenced Read Archive under accession numbers SAMD00632165–SAMD00632232. The activity and transcriptome data are available in Figshare (10.6084/m9.figshare.23672424 and 10.6084/m9.figshare.23672691, respectively).

### Supplementary information

Supplementary Information

Data set of cyclic transcripts
