# Supplementary material for: Plasticity of circadian and circatidal rhythms in activity and transcriptomic dynamics in a freshwater snail
Source: Heredity (Edinb). 2024 Mar 27;132(5):267–74. doi: 10.1038/s41437-024-00680-7 (PMC11074255; doi:10.1038/s41437-024-00680-7)
Supplement: Supplementary file 1 — Supplementary Information [file 41437_2024_680_MOESM1_ESM.docx]

**Supplementary Information for**

Plasticity of circadian and circatidal rhythms in activity and transcriptomic dynamics in a freshwater snail

Takumi Yokomizo and Yuma Takahashi

Corresponding author: Yuma Takahashi

Email: [takahashi.yum@gmail.com](mailto:takahashi.yum@gmail.com)


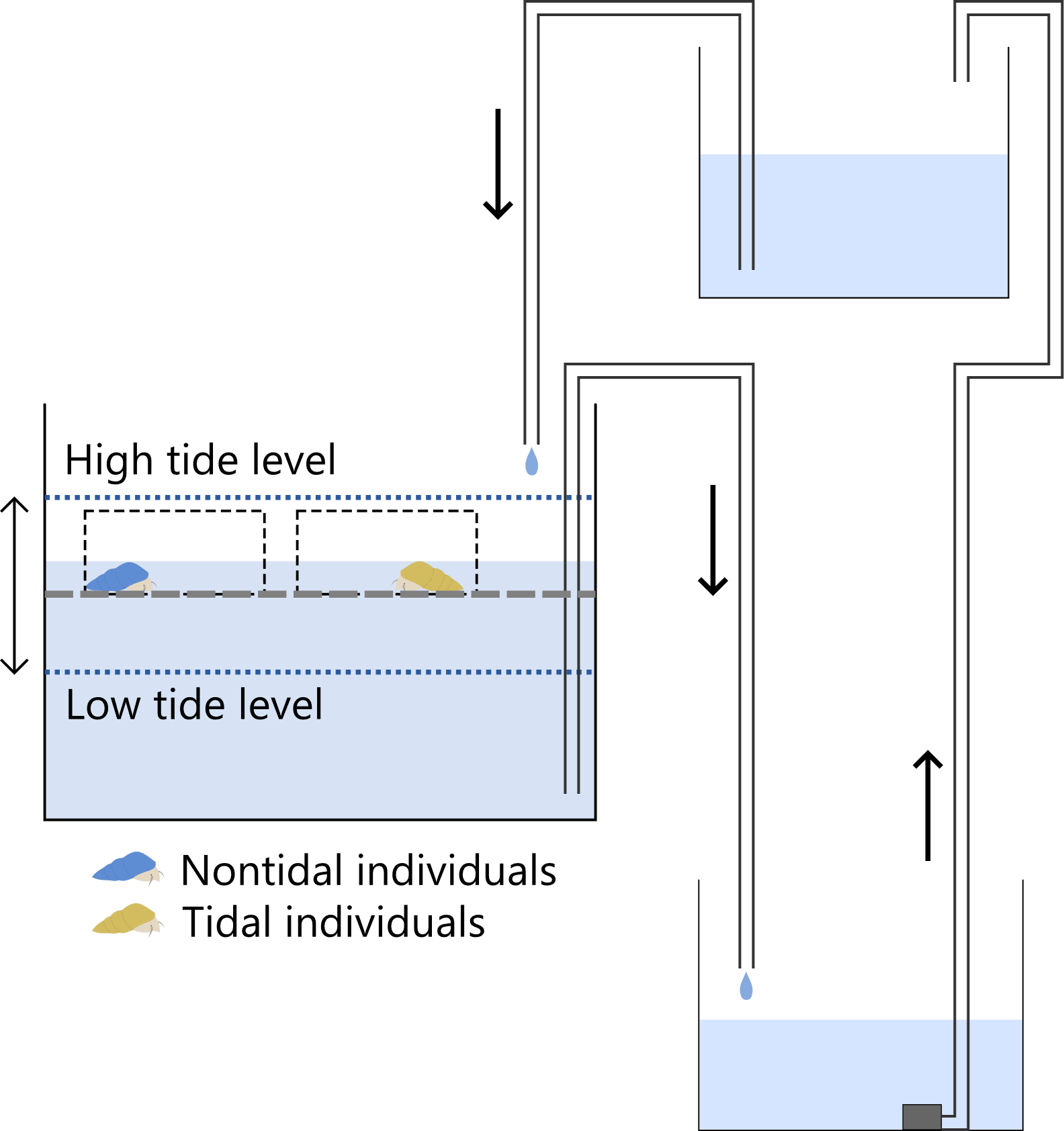


**Figure S1.** The structure of tidal simulation system. Snails were placed in the central tank (Main tank) and entrained to the tidal cycle. Water was supplied to and drained from the main tank using siphons and then pumped from the lower tank to the upper tank. In the main tank, snails were placed in the containers at the height of the dashed line. The dotted lines represent water levels of the high and low tides.


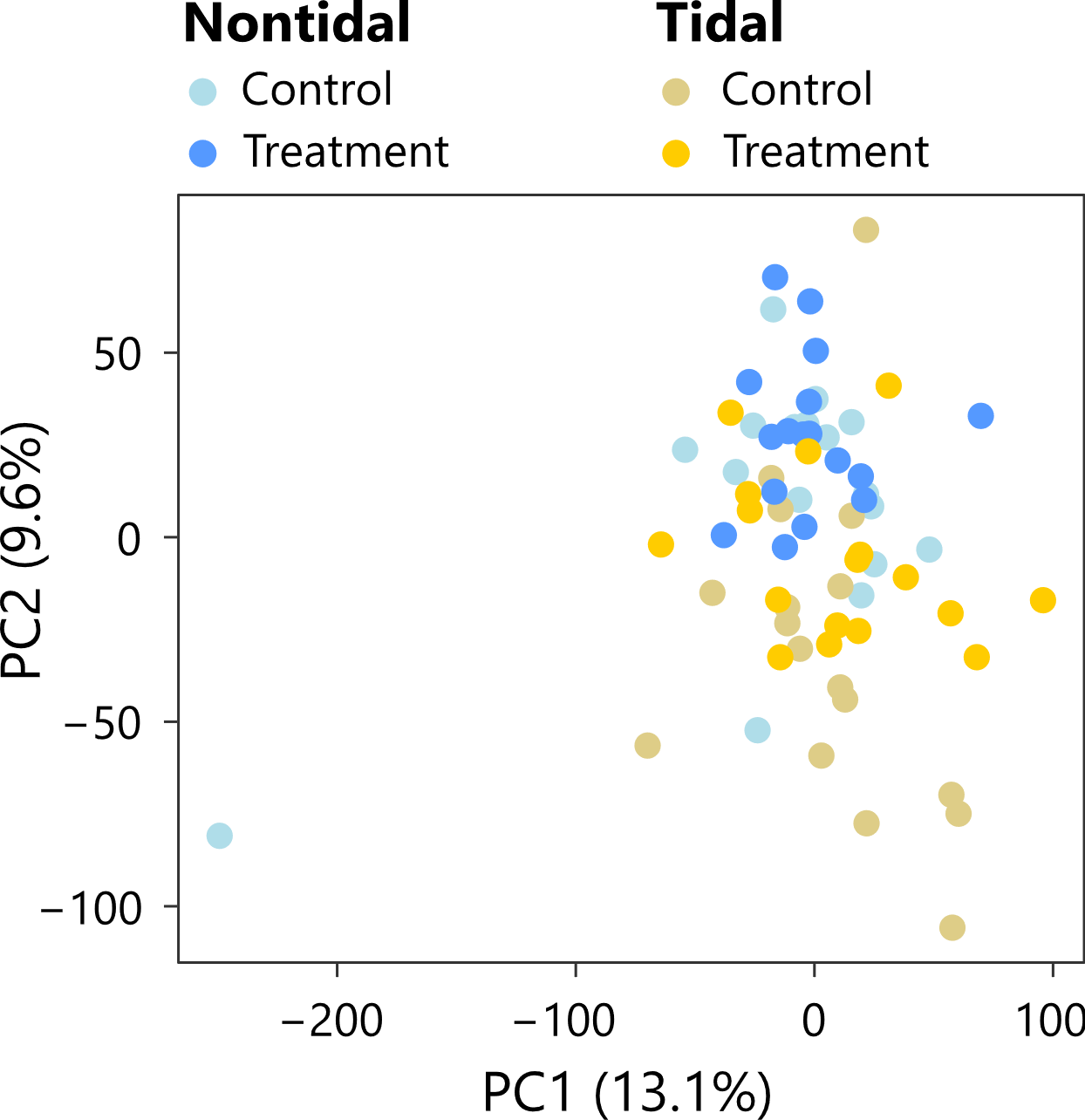


**Figure S2.** Probabilistic PCA of the expression of all genes after the filtering in nontidal and tidal populations using all samples.


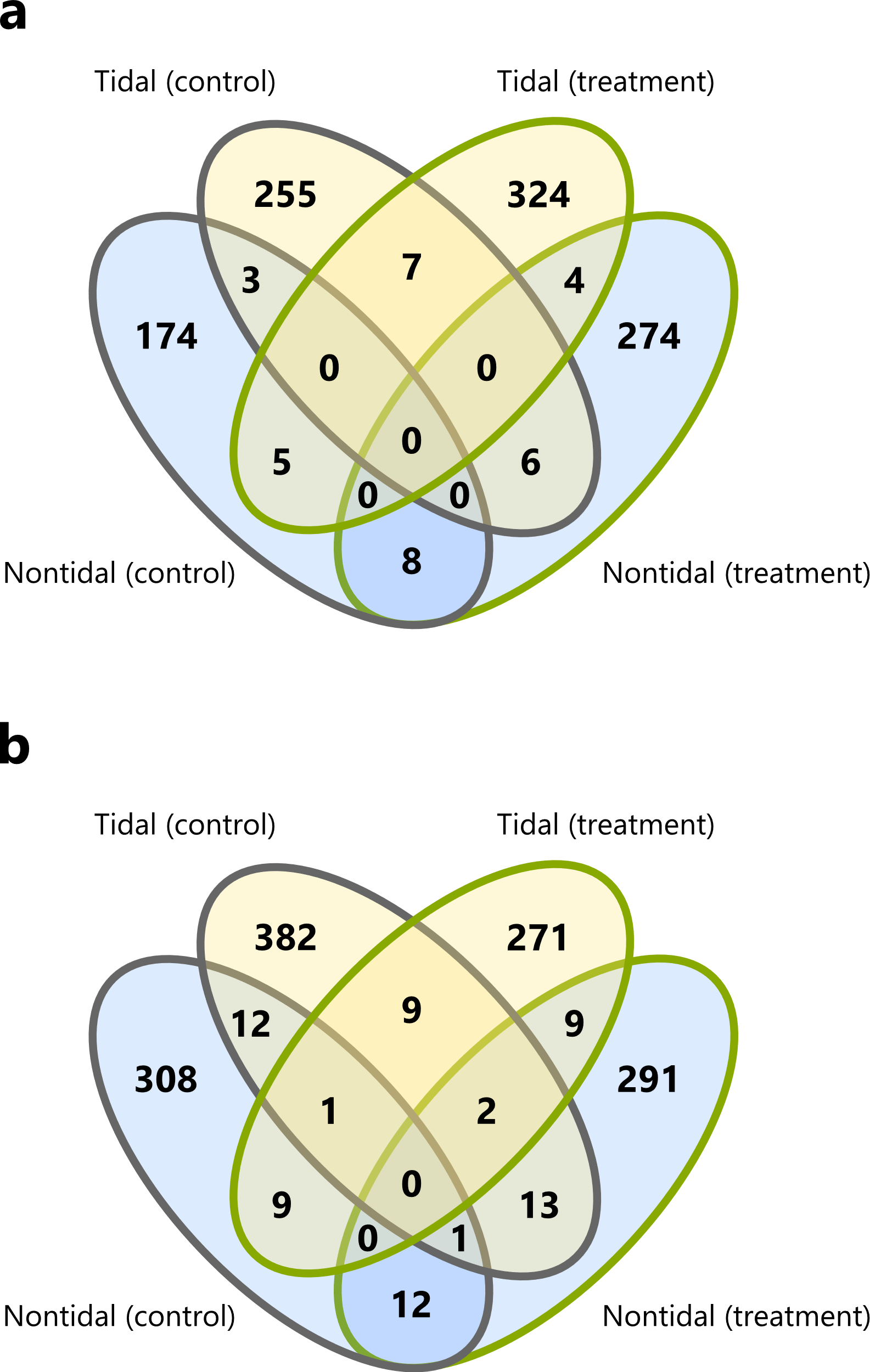


**Figure S3.** Venn diagrams for (a) circatidal and (b) circadian transcripts between tidal and nontidal populations.


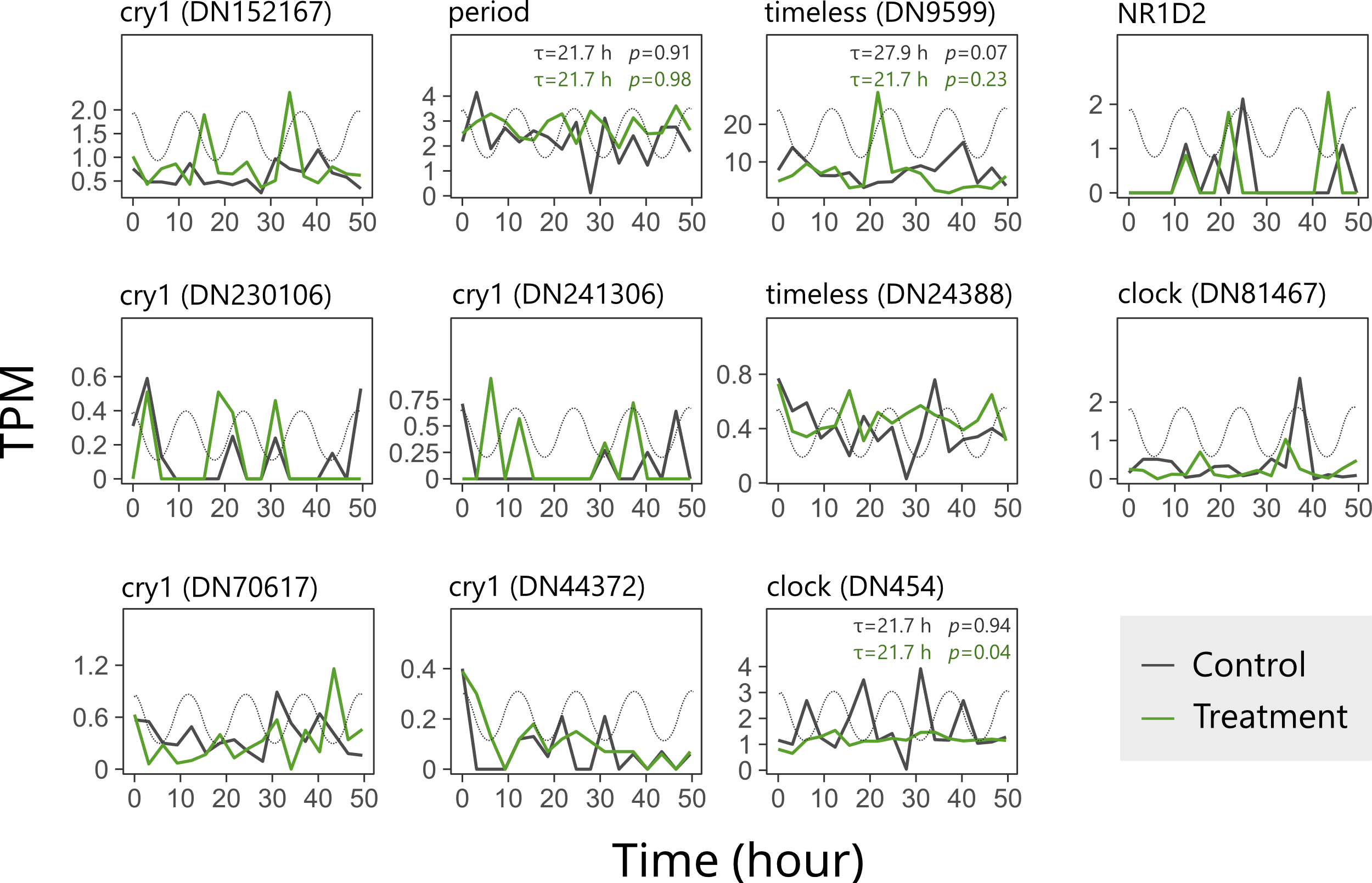


**Figure S4.** The expression patterns of circadian clock genes of the control (grey) and treatment (green) groups of the nontidal population under the DD condition. The simulated tidal cycle is shown as a grey dotted line. Genes which passed the filtering were analysed, and the p-value and period (τ) were shown.


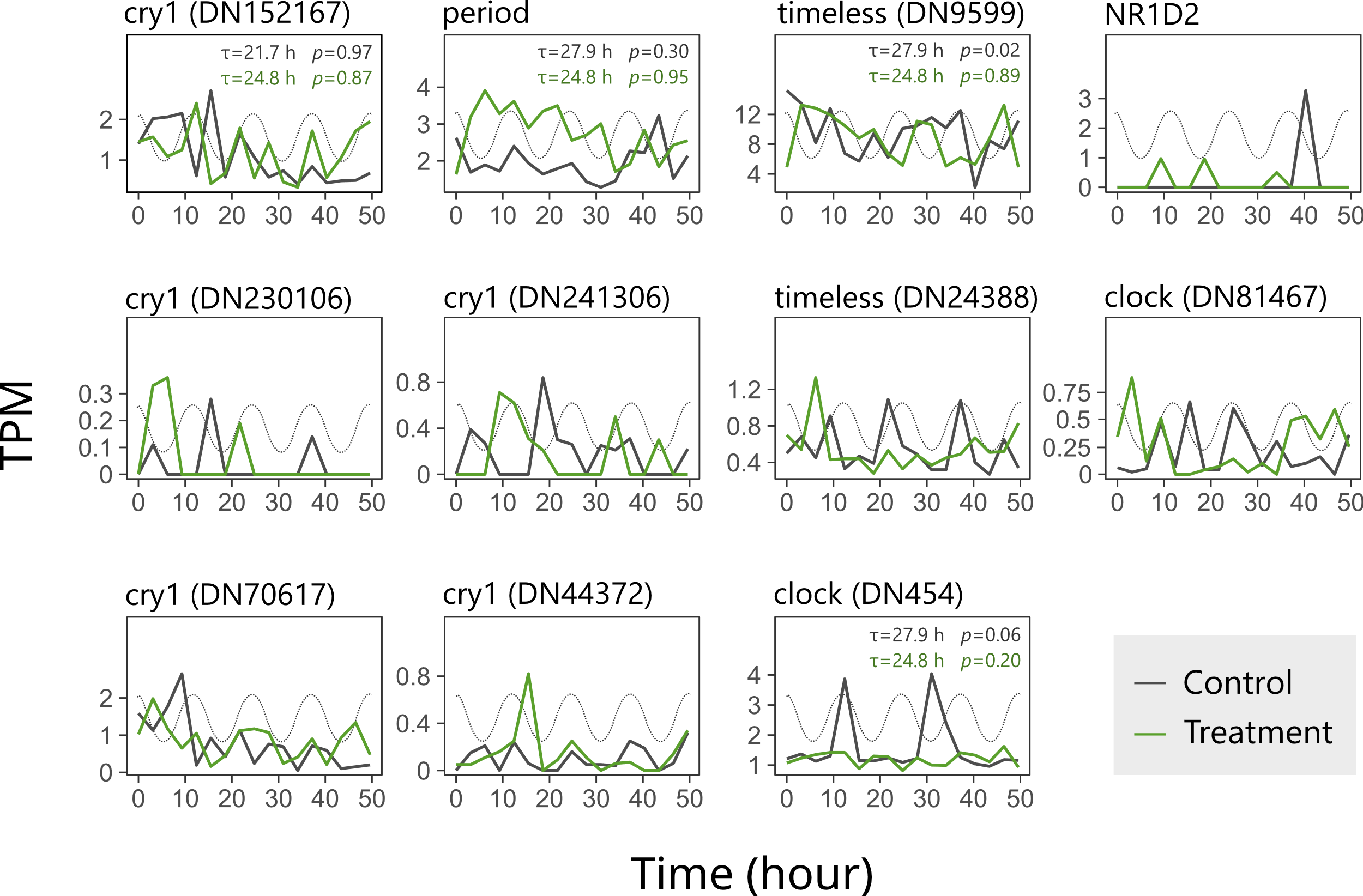


**Figure S5.** The expression patterns of circadian clock genes of the control (grey) and treatment (green) groups of the tidal population under the DD condition. The simulated tidal cycle is shown as a grey dotted line. Genes which passed the filtering were analysed, and the p-value and period (τ) were shown.


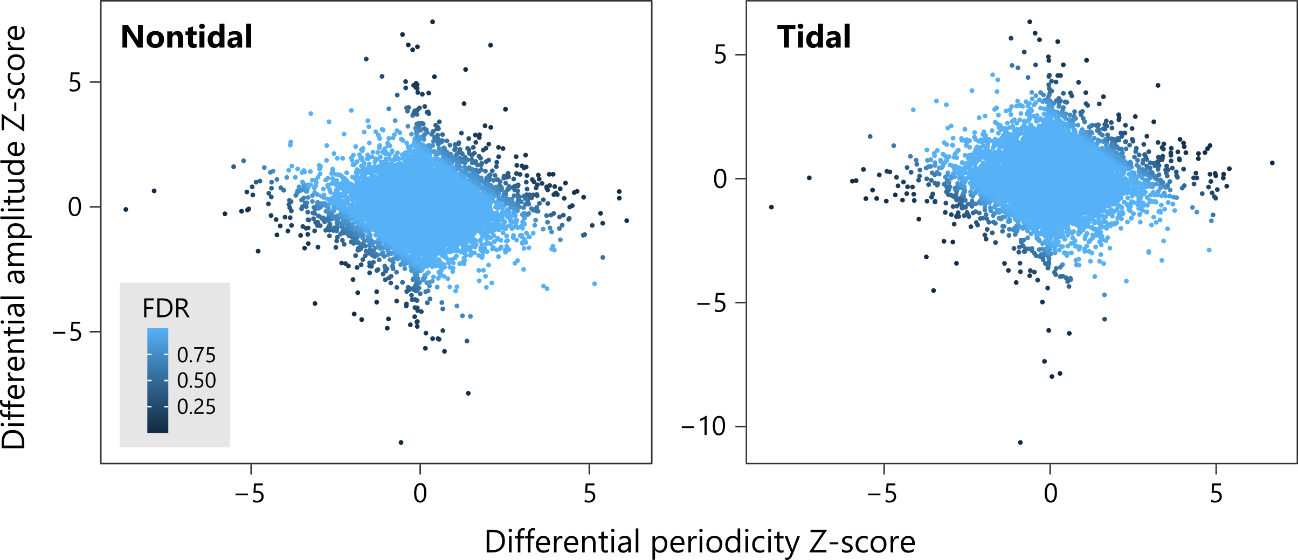


**Figure S6.** Changes in transcriptome rhythmicity between the control and treatment groups in the nontidal and tidal populations. The horizontal and vertical axis is *Z*-scores for changes in periodicity and amplitude between groups, respectively. Colours represent the FDR of *S*_DR_ for each gene.

**Table S1.** The number of raw reads in each sample.

| Sample | Population | Experiment | Time | Raw reads | High quality reads |
| --- | --- | --- | --- | --- | --- |
| mak_1c | Non-tidal | control | 0 | 11637217 | 10551537 |
| mak_2c | Non-tidal | control | 3.1 | 14517088 | 13159726 |
| mak_3c | Non-tidal | control | 6.2 | 12876528 | 11628906 |
| mak_4c | Non-tidal | control | 9.3 | 13158385 | 11888966 |
| mak_5c | Non-tidal | control | 12.4 | 12338172 | 11177784 |
| mak_6c | Non-tidal | control | 15.5 | 11384899 | 10286098 |
| mak_7c | Non-tidal | control | 18.6 | 13883070 | 12551939 |
| mak_8c | Non-tidal | control | 21.7 | 13259870 | 11942548 |
| mak_9c | Non-tidal | control | 24.8 | 13053790 | 11720442 |
| mak_10c | Non-tidal | control | 27.9 | 12874985 | 11616646 |
| mak_11c | Non-tidal | control | 31.0 | 13662857 | 12357711 |
| mak_12c | Non-tidal | control | 33.1 | 12385653 | 11174882 |
| mak_13c | Non-tidal | control | 37.2 | 11873204 | 10757085 |
| mak_14c | Non-tidal | control | 40.3 | 11778180 | 10642096 |
| mak_15c | Non-tidal | control | 43.4 | 11515721 | 10361132 |
| mak_16c | Non-tidal | control | 46.5 | 11516692 | 10420404 |
| mak_17c | Non-tidal | control | 49.6 | 12851196 | 11492932 |
| mak_18t | Non-tidal | treatment | 0 | 10738672 | 9229910 |
| mak_19t | Non-tidal | treatment | 3.1 | 10473783 | 8891001 |
| mak_20t | Non-tidal | treatment | 6.2 | 12469639 | 10923938 |
| mak_21t | Non-tidal | treatment | 9.3 | 11851404 | 10083989 |
| mak_22t | Non-tidal | treatment | 12.4 | 12567758 | 10958752 |
| mak_23t | Non-tidal | treatment | 15.5 | 12238422 | 10469730 |
| mak_24t | Non-tidal | treatment | 18.6 | 10183881 | 8865139 |
| mak_25t | Non-tidal | treatment | 21.7 | 13026176 | 11148177 |
| mak_26t | Non-tidal | treatment | 24.8 | 10178131 | 8722211 |
| mak_27t | Non-tidal | treatment | 27.9 | 14377518 | 12316403 |
| mak_28t | Non-tidal | treatment | 31.0 | 11302195 | 9649896 |
| mak_29t | Non-tidal | treatment | 33.1 | 10926096 | 9495603 |
| mak_30t | Non-tidal | treatment | 37.2 | 10994254 | 9542253 |
| mak_31t | Non-tidal | treatment | 40.3 | 10330389 | 8960229 |
| mak_32t | Non-tidal | treatment | 43.4 | 12231522 | 10495053 |
| mak_33t | Non-tidal | treatment | 46.5 | 13839002 | 11861922 |
| mak_34t | Non-tidal | treatment | 49.6 | 10985898 | 9530984 |
| tat_1c | Tidal | control | 0 | 14193317 | 13083139 |
| tat_2c | Tidal | control | 3.1 | 14707953 | 13605485 |
| tat_3c | Tidal | control | 6.2 | 13724581 | 12582400 |
| tat_4c | Tidal | control | 9.3 | 12917632 | 11801816 |
| tat_5c | Tidal | control | 12.4 | 14769246 | 13511967 |
| tat_6c | Tidal | control | 15.5 | 11551791 | 10539025 |
| tat_7c | Tidal | control | 18.6 | 12673541 | 11659424 |
| tat_8c | Tidal | control | 21.7 | 12343878 | 11293300 |
| tat_9c | Tidal | control | 24.8 | 13471394 | 12333511 |
| tat_10c | Tidal | control | 27.9 | 12894287 | 11874765 |
| tat_11c | Tidal | control | 31.0 | 13922733 | 12752476 |
| tat_12c | Tidal | control | 33.1 | 16295868 | 14898727 |
| tat_13c | Tidal | control | 37.2 | 11858458 | 10824923 |
| tat_14c | Tidal | control | 40.3 | 11634813 | 10626164 |
| tat_15c | Tidal | control | 43.4 | 13437996 | 12278517 |
| tat_16c | Tidal | control | 46.5 | 13235442 | 11964217 |
| tat_17c | Tidal | control | 49.6 | 13847547 | 12544824 |
| tat_18t | Tidal | treatment | 0 | 13584585 | 12413127 |
| tat_19t | Tidal | treatment | 3.1 | 15781059 | 14377144 |
| tat_20t | Tidal | treatment | 6.2 | 14143894 | 12921721 |
| tat_21t | Tidal | treatment | 9.3 | 13128863 | 11964191 |
| tat_22t | Tidal | treatment | 12.4 | 11621822 | 10575967 |
| tat_23t | Tidal | treatment | 15.5 | 11531164 | 10500362 |
| tat_24t | Tidal | treatment | 18.6 | 12843546 | 11727401 |
| tat_25t | Tidal | treatment | 21.7 | 16007631 | 14594872 |
| tat_26t | Tidal | treatment | 24.8 | 15515239 | 14208064 |
| tat_27t | Tidal | treatment | 27.9 | 12808593 | 11687999 |
| tat_28t | Tidal | treatment | 31.0 | 12518777 | 11321597 |
| tat_29t | Tidal | treatment | 33.1 | 11375355 | 10290611 |
| tat_30t | Tidal | treatment | 37.2 | 10926448 | 9827267 |
| tat_31t | Tidal | treatment | 40.3 | 10411385 | 9434878 |
| tat_32t | Tidal | treatment | 43.4 | 13037979 | 11795702 |
| tat_33t | Tidal | treatment | 46.5 | 11425600 | 10410942 |
| tat_34t | Tidal | treatment | 49.6 | 11877840 | 10756629 |

**Table S2.** Genes showing significant oscillation of the circadian period in the control group and the circatidal period in the treatment group.

| Transcripts | population | Description |
| --- | --- | --- |
| DN1614_c0_g1 | Nontidal | rRNA methyltransferase 3, mitochondrial isoform X2 |
| DN1744_c50_g1 | Nontidal | uncharacterized protein |
| DN242957_c0_g2 | Nontidal | uncharacterized protein |
| DN123481_c0_g1 | Nontidal | zinc finger protein 665 |
| DN13546_c3_g1 | Nontidal | uncharacterized protein |
| DN3297_c6_g1 | Nontidal | neuroglian isoform X1 |
| DN12146_c0_g1 | Nontidal | uncharacterized protein |
| DN1746_c0_g3 | Tidal | uncharacterized protein |
| DN8167_c0_g1 | Tidal | cytochrome c oxidase assembly factor 5-like |
| DN18033_c1_g1 | Tidal | uncharacterized protein |
| DN2460_c3_g1 | Tidal | thrombospondin type-1 domain-containing protein 4 |
| DN2825_c0_g1 | Tidal | uncharacterized protein |
| DN3415_c3_g1 | Tidal | mitochondrial inner membrane protein OXA1L |
| DN1804_c0_g1 | Tidal | probable 18S rRNA (guanine-N(7))-methyltransferase |
| DN29026_c0_g1 | Tidal | adenine DNA glycosylase |

**Table S3.** Genes that significantly increased rhythmicity of the circatidal period in the treatment group of the nontidal population. FDR is computed for *S*_DR_ using a Gaussian distribution based on the fit to the empirical distribution.

| Transcripts | *S*_DR_ | FDR | Description |
| --- | --- | --- | --- |
| DN21673_c2_g1 | 6.03 | 3.0 × 10^−6^ | transcription elongation factor 1 homolog isoform X2 |
| DN192135_c0_g1 | 5.48 | 6.7 × 10^−5^ | TIP41-like protein isoform X2 |
| DN449_c2_g1 | 4.82 | 0.0015 | uncharacterized protein |
| DN105095_c2_g2 | 4.58 | 0.0039 | uncharacterized protein |
| DN207785_c0_g1 | 4.53 | 0.0045 | uncharacterized protein |
| DN39152_c0_g1 | 4.49 | 0.0050 | neurogenic locus notch homolog protein 2 isoform X17 |
| DN2536_c14_g1 | 4.46 | 0.0054 | sorting nexin-14 isoform X1 |
| DN18313_c0_g1 | 4.40 | 0.0058 | uncharacterized protein |
| DN2970_c2_g2 | 4.32 | 0.0079 | tRNA (adenine(58)-N(1))-methyltransferase non-catalytic subunit TRM6 |
| DN6087_c0_g1 | 4.27 | 0.0090 | kinesin-like protein KIF21A isoform X14 |
| DN53630_c0_g1 | 3.97 | 0.030 | ABC transporter F family member 4 isoform X2 |
| DN93751_c0_g1 | 3.92 | 0.036 | multiple epidermal growth factor-like domains protein 10 isoform X3 |
| DN30054_c0_g1 | 3.83 | 0.045 | dual adapter for phosphotyrosine and 3-phosphotyrosine and 3-phosphoinositide isoform X3 |

**Table S4.** Genes that significantly increased rhythmicity of the circatidal period in the treatment group of the tidal population. FDR is computed for *S*_DR_ using a Gaussian distribution based on the fit to the empirical distribution.

| Transcripts | *S*_DR_ | FDR | Description |
| --- | --- | --- | --- |
| DN19687_c0_g1 | 5.16 | 2.9 × 10^−4^ | ribonuclease P protein subunit p29 |
| DN50332_c0_g1 | 4.94 | 7.5 × 10^−4^ | uncharacterized protein |
| DN6135_c0_g1 | 4.33 | 0.0098 | uncharacterized protein |
| DN702_c1_g1 | 4.19 | 0.015 | glutaminyl-peptide cyclotransferase |
| DN7_c3_g1 | 4.13 | 0.018 | uncharacterized protein |
| DN10387_c0_g1 | 4.08 | 0.020 | S-adenosylmethionine synthase |
| DN210683_c0_g1 | 4.05 | 0.021 | solute carrier family 35 member F5 isoform X3 |
| DN148503_c0_g1 | 4.02 | 0.022 | CD209 antigen-like protein E |
| DN48586_c2_g1 | 4.02 | 0.022 | uncharacterized protein |
| DN140387_c0_g1 | 3.87 | 0.036 | uncharacterized protein |
| DN67461_c2_g1 | 3.86 | 0.037 | zinc finger protein 665 |
| DN312_c13_g1 | 3.84 | 0.038 | uncharacterized protein |
| DN177547_c2_g1 | 3.81 | 0.040 | uncharacterized protein |
| DN6762_c5_g1 | 3.81 | 0.040 | uncharacterized protein |
| DN618_c4_g2 | 3.80 | 0.041 | uncharacterized protein |
| DN7859_c1_g1 | 3.75 | 0.047 | transcription elongation factor A N-terminal and central domain-containing protein 2-like |
